# Acute Psychological Stress Modulates the Expression of Enzymes Involved in the Kynurenine Pathway throughout Corticolimbic Circuits in Adult Male Rats

**DOI:** 10.1155/2016/7215684

**Published:** 2015-12-27

**Authors:** Haley A. Vecchiarelli, Chaitanya P. Gandhi, Matthew N. Hill

**Affiliations:** ^1^Hotchkiss Brain Institute, Cumming School of Medicine, University of Calgary, 3330 Hospital Drive NW, Calgary, AB, Canada T2N 4N1; ^2^Mathison Centre for Mental Health Research and Education, Cumming School of Medicine, University of Calgary, 3330 Hospital Drive NW, Calgary, AB, Canada T2N 4N1; ^3^Department of Neuroscience, Cumming School of Medicine, University of Calgary, 3330 Hospital Drive NW, Calgary, AB, Canada T2N 4N1; ^4^Department of Cell Biology and Anatomy, Cumming School of Medicine, University of Calgary, 3330 Hospital Drive NW, Calgary, AB, Canada T2N 4N1; ^5^Department of Psychiatry, Cumming School of Medicine, University of Calgary, 3330 Hospital Drive NW, Calgary, AB, Canada T2N 4N1

## Abstract

Tryptophan is an essential dietary amino acid that is necessary for protein synthesis, but also serves as the precursor for serotonin. However, in addition to these biological functions, tryptophan also serves as a precursor for the kynurenine pathway, which has neurotoxic (quinolinic acid) and neuroprotective (kynurenic acid) metabolites. Glucocorticoid hormones and inflammatory mediators, both of which are increased by stress, have been shown to bias tryptophan along the kynurenine pathway and away from serotonin synthesis; however, to date, there is no published data regarding the effects of stress on enzymes regulating the kynurenine pathway in a regional manner throughout the brain. Herein, we examined the effects of an acute psychological stress (120 min restraint) on gene expression patterns of enzymes along the kynurenine pathway over a protracted time-course (1–24 h post-stress termination) within the amygdala, hippocampus, hypothalamus, and medial prefrontal cortex. Time-dependent changes in differential enzymes along the kynurenine metabolism pathway, particularly those involved in the production of quinolinic acid, were found within the amygdala, hypothalamus, and medial prefrontal cortex, with no changes seen in the hippocampus. These regional differences acutely may provide mechanistic insight into processes that become dysregulated chronically in stress-associated disorders.

## 1. Introduction

Tryptophan is an essential dietary amino acid. It is well known that it serves as the precursor for serotonin, but what is less well known is that the majority (90–95%) of tryptophan besides that utilized for protein synthesis, is shuttled to the kynurenine pathway, the end point of which is the generation of nicotinamide adenine dinucleotide (NAD+). Metabolites along the kynurenine pathway both are synthesized in the brain and can travel into the brain [1]. Furthermore, upregulation of the kynurenine pathway has been demonstrated in depressive disorders, and the intensity of depressive symptoms has been found to correlate with the upregulation of kynurenine pathway [2–10]. The enhancement of this differential metabolic route of tryptophan has been hypothesized to contribute to potential alterations in serotonergic function in depression, such that an increase shuttling of tryptophan to the kynurenine pathway could result in a deficit in serotonin synthesis [11].

**Figure 1:**
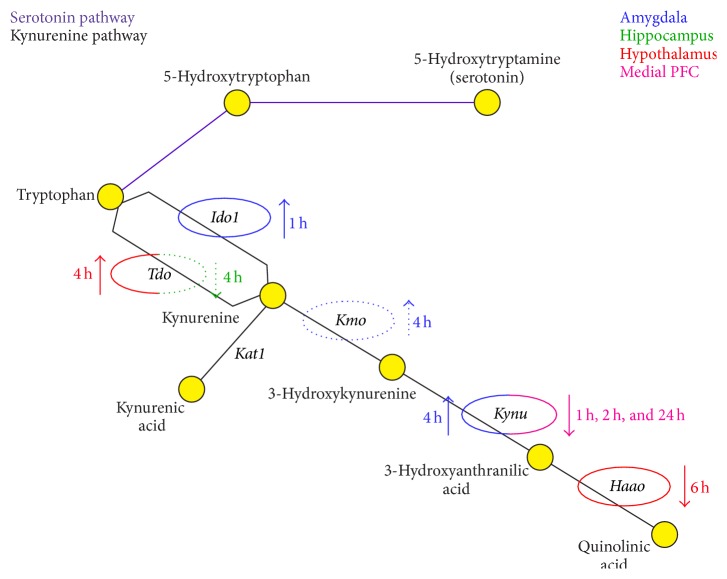
Schematic representation of tryptophan metabolism and its influence by stress. This figure shows the multiple ways that tryptophan can be metabolized. The classic, well-known pathway is its conversion to serotonin (purple). The primary pathway of tryptophan metabolism, however, is the kynurenine pathway (black), by which tryptophan is converted to kynurenine via tryptophan 2,3-dioxygenase (TDO) or indoleamine 2,3-dioxygenase 1 (IDO1). From there, it can be converted into the neuroprotective kynurenic acid (KNYA) via kynurenine aminotransferase (KAT1) or to quinolinic acid (QUIN) via a multistep process involving the enzymes kynurenine 3-monooxygenase (KMO), kynureninase (KYNU), and 3-hydroxyanthranilate 3,4-dioxygenase (3-HOA). An analysis of mRNA expression levels of tryptophan 2,3-dioxygenase (*Tdo*), indoleamine 2,3-dioxygenase 1 (*Ido1*), kynurenine aminotransferase (*Kat1*), kynurenine 3-monooxygenase (*Kmo*), kynureninase (*Kynu*), and 3-hydroxyanthranilate 3,4-dioxygenase (*Haao*) revealed increases in* Ido1*,* Kmo*, and* Kynu* mRNA expression in the amygdala (blue), decreases in* Tdo* mRNA expression in the hippocampus (green), an upregulation of* Tdo*, and downregulation of* Haao* mRNA expression in the hypothalamus (red) and a downregulation of* Kynu* mRNA expression in the medial prefrontal cortex (pink) (solid lines indicate significant changes, while dashed lines indicate trending changes). This figure summates the regional and temporal effects of acute stress on the mRNA expression of kynurenine pathway enzymes.

The rate-limiting step in this pathway is the conversion of tryptophan to kynurenine by either tryptophan 2,3-dioxygenase (TDO) or indoleamine 2,3-dioxygenase 1 (IDO1). Kynurenine can then be metabolized along a variety of pathways. One pathway results in the conversion of kynurenine to kynurenic acid (KYNA) by kynurenine aminotransferase1 (KAT1). KYNA is an N-methyl-D-aspartate receptor (NMDAR) and *α*7 nicotinic acetylcholine receptor (*α*7nAChR) antagonist and is thought to play a neuroprotective role through inhibiting glutamate signaling [1]. The other pathway, which results in the generation of quinolinic acid (QUIN), has multiple steps, by which kynurenine is converted to intermediates through kynurenine 3-monooxygenase (KMO) and kynureninase (KYNU) and, finally, by 3-hydroxyanthranilic acid 3,4-dioxygenase (3-HOA) to QUIN (Figure 1) [1]. As a highly selective NMDAR agonist, QUIN can induce excitotoxicity [1]. When taken altogether, the balance of the kynurenine metabolites, particularly KYNA and QUIN, could play an important role in central nervous system functioning, and that disruption of this balance could be important for a number of neurological disorders, including neurodegenerative and affective disorders [1].

It has been well established that inflammation can affect the activity of enzymes regulating the kynurenine pathway [12]. Specifically, IDO1 is upregulated by proinflammatory cytokines, such as TNF-*α* and IFN*γ*, and it has also been shown to be downregulated by anti-inflammatory cytokines, such as IL-4 [12]. Furthermore, there is evidence that stress, and specifically the major output of the HPA axis, glucocorticoids, can upregulate TDO [12]. Taken together, these data suggest that stress and/or inflammation have the ability to upregulate levels of kynurenine. Consistent with this, kynurenine levels are elevated in diseases that have elevated glucocorticoid output and inflammatory mediators, such as major depression and coronary heart disease [1, 2]. This increased pool of kynurenine may serve as a precursor for the generation of downstream metabolites, and in the context of disease states, it may amplify the production of the neurotoxic QUIN, which has been hypothesized to contribute to structural alterations and loss of neuropil in disease states such as major depression [8, 12, 13].

It is now well established that acute stress upregulates inflammatory mediators, both in humans, peripherally, and in rodents, both peripherally and in the brain [14, 15]. Recent work from our group has demonstrated that stress-induced changes in inflammatory mediators within the brain are not uniform and exhibit significant spatiotemporal specificity, with the amygdala appearing to be highly sensitive to induction of proinflammatory molecules following exposure to acute psychological stress [16]. Given that stress increases inflammatory cytokines and glucocorticoid levels, both of which have been shown to increase kynurenine levels, it seems highly plausible that stress could exert robust and dynamic effects on the pathway regulating kynurenine synthesis. Consistent with this hypothesis, it has been shown that various acute stressors can lead to an upregulation of the kynurenine pathway [17–19]; however, only one of these studies actually examined expression levels of the enzymes involved in kynurenine metabolism and did so in a whole brain manner without any regional specificity [17]. Therefore, the goal of this study was to investigate whether acute stress exposure affected gene expression levels of the enzymes in the kynurenine pathway, particularly investigating the key corticolimbic brain regions involved in regulating the stress response, the amygdala, hippocampus, hypothalamus, and medial prefrontal cortex, in a time-dependent manner.

## 2. Materials and Methods

### 2.1. Animals

Adult (approximately 70 postnatal days old, 300 g), male, Sprague Dawley rats (*N* = 48), obtained from Charles River (St. Constant, QC, Canada), were used in the following experiment. Animals were allowed to acclimate for at least one week prior to testing and kept on a 12:12 hour (h) light/dark cycle with* ad libitum* access to food and water. The experiment was conducted during the light phase of the cycle. The University of Calgary Animal Care Committee and the Canadian Council for Animal Care approved all animal use.

### 2.2. Experimental Design

Animals were subjected to 120 minutes (min) of restraint stress within the first 2 h of the light cycle, using clear, Plexiglas, Broome Style restrainers (Plas-Labs, Lansing, MI, United States). Rats were then decapitated 1 h, 2 h, 4 h, 6 h, or 24 h following stress termination (*n* = 6 for all time points). A nonstressed basal group (*n* = 6), designated as B, was implemented to allow for comparison of relative mRNA expression. However, given that circadian variations in inflammatory mediators exist in rats [20–22], two additional basal groups, referred to as 4B and 6B, were implemented for the 4 h (*n* = 6) and 6 h (*n* = 6) time points. Hence, there were three control groups: B, 4B, and 6B, which were used as controls for animals in the 1 h, 2 h, and 24 h groups, the 4 h group, and the 6 h group, respectively.

Following animal sacrifice, trunk blood was collected in Vacutainer blood collection tubes containing K2 ethylenediaminetetraacetic acid (EDTA) (BD, Mississauga, ON, Canada) and placed on ice for a maximum of 30 min before centrifugation at 10,000 g and 4°C for 20 min. Plasma was aliquoted and stored at −80°C for the measurement of corticosterone. Brains were extracted and the amygdala, hippocampus, hypothalamus, and medial prefrontal cortex were dissected out as previously described [23]. Following dissection, brain regions were immediately flash frozen on dry ice and stored in 2 mL RNase-free microtubes (Diamed, Mississauga, ON, Canada) at −80°C for further processing. Tissue collection areas and tools were sterilized with 70% ethanol and RNaseZAP (Sigma-Aldrich, Oakville, ON, Canada) between dissections to prevent RNase activity.

### 2.3. Corticosterone ELISA

Plasma corticosterone levels were measured in duplicate using a commercially available enzyme linked immunosorbent assay (ELISA) kit (Cayman Chemical Company, Ann Arbor, MI, United States), according to the manufacturer's protocol. The limit of detection for this assay at 80% binding/maximal binding is 30 pg/mL, while the standard curve ranges from 8.3 pg/mL to 5,000 pg/mL. Intra- and interassay variability are less than 10% at the middle points of the standard curve. All plasma samples were diluted 1 : 1000 so that values fell within the standard curve.

### 2.4. Tissue Processing for mRNA Isolation and cDNA Synthesis

Throughout the entirety of the mRNA isolation procedure, 70% ethanol and RNaseZAP (Sigma-Aldrich, Oakville, ON, Canada) were used to ensure that contamination by RNases did not occur. mRNA was isolated from each sample using the RNeasy Plus Universal Mini kit (Qiagen, Toronto, ON, Canada) and the QIAcube (Qiagen), according to the manufacturer's protocols. Briefly, samples were placed in 900 *μ*L of QIAzol lysis reagent (Qiagen), containing phenol and guanidine thiocyanate, and homogenized for 2 min at 50 Hz using 50 mm steel beads (Qiagen) with a TissueLyser LT homogenizer (Qiagen). After lysis, 100 *μ*L of gDNA eliminator solution and 180 *μ*L of chloroform were added to the samples, followed by centrifugation for 15 min at 4°C and 12,000 g. The aqueous phase containing mRNA was transferred to new microcentrifuge tubes and placed in the QIAcube to carry out the RNeasy Plus Universal program. In this automated protocol, 600 *μ*L of 70% ethanol is added to the samples, which are transferred to spin columns and centrifuged for 15 sec at 20°C and 8,000 g. The centrifugation process is repeated and followed by buffer rinses, to aid in the removal of non-mRNA biomolecules. Finally, the spin column is placed in a 1.5 mL collection tube and nuclease-free H_2_O (Qiagen) was added to the samples so that they were eluted to a final volume of 100 *μ*L. A Nanodrop spectrophotometer (Thermo Fisher Scientific, Waltham, MA, United States) was used to measure mRNA concentration and purity (using the* A*_260_/*A*_280_ ratio). Samples were then aliquoted and frozen at −80°C. All samples had an* A*_260_/*A*_280_ ratio value between 1.9 and 2.1, indicating high mRNA purity.

In order to synthesize cDNA, qScript cDNA SuperMix (Quanta Biosciences, Gaithersburg, MD, United States) was used, according to manufacturer's protocols. To summarize, 1 *μ*g of mRNA was added to 4 *μ*L of a reaction buffer (MgCl_2_, dNTPs, recombinant RNase inhibitor protein, qScript reverse transcriptase, random primers, oligo(dT) primer, and stabilizers) and nuclease-free H_2_O to result in a solution with a final volume of 20 *μ*L. Samples were then incubated for 5 min at 25°C, 30 min at 42°C, and 5 min at 85°C and held at 4°C. As with mRNA isolation, cDNA samples were read on a Nanodrop spectrophotometer to measure cDNA content and stored at −20°C. Prior to quantitative PCR (qPCR) analysis, cDNA aliquots were diluted in nuclease-free H_2_O to a final concentration of 25 ng/*μ*L.

### 2.5. Quantitative PCR

Primers were first designed using the IDTDNA software (Coralville, IA, United States), PrimerQuest. Although all efforts were taken to check the literature for duplication of previous primers, any replication of previously published primers is purely accidental and likely due to using the same software program. mRNA sequences for genes of interest were obtained using NCBI Nucleotide searches. The program was then instructed to construct primers that would result in products of no longer than 200 base pairs (bp) and had annealing temperatures (*T*_*m*_) of 52–55°C. Primer pairs that met these criteria were then analyzed with the NCBI BLAST tool to check for specificity. Finally, primers were reconstituted and diluted in nuclease-free H_2_O to obtain a final stock concentration of 10 *μ*M. For a list of primer sequences, see Table 1.

Quantitative PCR (qPCR) was performed using the Quanta PerfeCTa SYBR Green FastMix (Quanta Biosciences) on a Rotor-Gene Q (Qiagen), according to manufacturer's instructions. Briefly, 50 ng of cDNA, primers (final concentration of 1 *μ*M for forward and reverse each), and nuclease-free H_2_O were added to a reaction buffer (MgCl_2_, dNTPs, AccuFast Taq DNA Polymerase, SYBR Green I dye, and stabilizers). First, each primer was analyzed in a serially diluted standard curve to assess proper reaction conditions (number of cycles and annealing temperature) and to check for primer reaction efficiency (must be between 80 and 120%). All primers were assayed using the following specifications: 1 min at 90°C, 40 cycles of 95°C for 5 sec and 60°C for 30 sec. This was followed by a melt step (72°C to 95°C) to assess for single primer products. Each gene of interest's primer pair was compared with all reference primer pairs to ascertain which ones have the most similar reaction efficiencies. For each gene of interest, one reference primer was chosen that matched its efficiency.

Since all samples per brain region could not fit in one qPCR assay, they were divided as follows: assays contained at least one sample from the B, 1 h, 2 h, and 24 h groups, from the 4B and 4 h group, or from the 6B and 6 h group. For each run, a standard curve for each primer was run to continually validate primer efficiencies. Likewise, for each sample, control primers and primers of interest were assayed in triplicate, while no template controls (containing nuclease-free H_2_O) were assayed singularly.

The *C*_*t*_ value, or the point at which fluorescent detection crosses the threshold, was assessed for each well. The three *C*_*t*_ values for each sample were averaged for both the reference primer and primer of interest. The difference between the reference primer and primer of interest, Δ*C*_*t*_, was calculated for each sample. Subsequently, the difference between the average Δ*C*_*t*_ value for the basal groups (B, 4B, and 6B) and each sample (from the 1 h, 2 h, 24 h, 4 h, and 6 h groups), ΔΔ*C*_*t*_, was calculated. Finally, the 2^−ΔΔ*C*_*t*_^ values for each sample were calculated and the data was normalized so that the averages 2^−ΔΔ*C*_*t*_^ of the basal groups were 1.

### 2.6. Statistical Analysis

All data were analyzed using GraphPad Prism software (GraphPad, La Jolla, CA, United States). Before any analyses were performed, outliers were removed using the robust regression and outlier removal (ROUT) method in the software. It involves using nonlinear regression to fit a curve that is not influenced by outliers [25]. This is followed by the identification of outliers using the residuals of the robust fit.

One-way analyses of variance (ANOVA), and if applicable* post hoc* analyses with Bonferroni's correction for multiple comparisons, was used to compare the means of the 1 h, 2 h, and 24 h groups to the B group, while two-tailed Student's* t*-tests were used to compare the relative mRNA expression levels of the 4 h and 6 h groups to the 4B and 6B group, respectively. *F*- or* t*-values, *p*-values, and eta square (*R* ^2^) values are reported for all data. All data are reported as means ± standard error of the mean (SEM) and *p* < 0.05 was considered the threshold for statistical significance.

## 3. Results

### 3.1. Effects of Acute Psychological Stress on Plasma Corticosterone Concentrations

Two hours of restraint stress reliably elevates plasma corticosterone in our hands [16], but to determine if stress had residual effects on plasma corticosterone at the time points we examined gene expression, we measured plasma corticosterone levels at all time points. Plasma corticosterone levels were not altered 1 h, 2 h, or 24 h post-stress termination (*F*(3,19) = 1.75, *p* > 0.05, and *R* ^2^ = 0.22), compared to their time-matched basal (B) group (Table 2). Similarly, corticosterone levels remained the same at both the 4 h (*t*(9) = 2.15, *p* > 0.05, and *R* ^2^ = 0.34) and 6 h time points post-stress termination (*t*(9) = 0.17, *p* > 0.05, and *R* ^2^ = 0.003), relative to their time-matched controls (Table 2). Finally, two-tailed* t*-tests showed a significant increase in the 6B group compared to the B group (*t*(10) = 2.60, *p* = 0.03, and *R* ^2^ = 0.40) but not between the 4B and B (*t*(10) = 0.72, *p* > 0.05, and *R* ^2^ = 0.05), which is indicative of circadian changes in basal levels of corticosterone (Table 2). This suggests that stress-induced corticosterone levels had recovered to basal levels at all time points examined in this study.

### 3.2. Acute Psychological Stress Upregulates* Ido1*,* Kmo*, and* Kynu* mRNA Expression in the Amygdala

**Figure 2:**
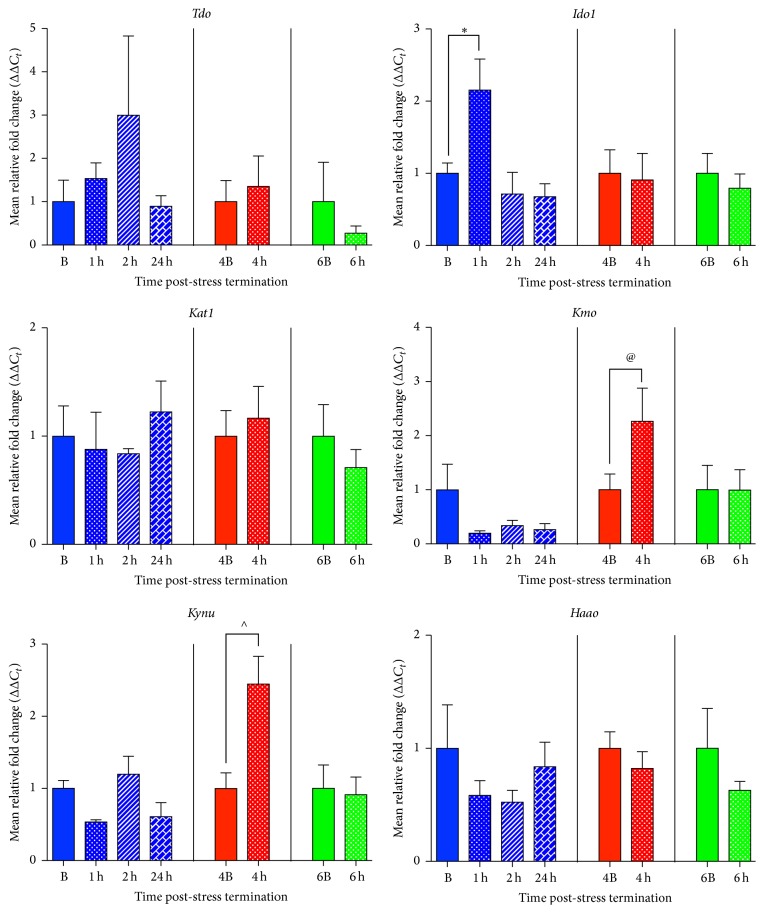
Effects of acute restraint stress on enzymes regulating the kynurenine pathway in the amygdala. mRNA expression levels of tryptophan 2,3-dioxygenase (*Tdo*), indoleamine 2,3-dioxygenase 1 (*Ido1*), kynurenine aminotransferase1 (*Kat1*), kynurenine 3-monooxygenase (*Kmo*), kynureninase (*Kynu*), and 3-hydroxyanthranilate 3,4-dioxygenase (*Haao*) were examined 1–24 h post-stress termination. All data are represented as means ± SEM (*n* = 3–6/group). ^*∗*^ *p* < 0.05 (in comparison to the B group), ^∧^ *p* < 0.05 (in comparison to the 4B group), and ^@^ *p* < 0.10 (in comparison to the 4B group).

120 min of restraint stress altered amygdalar mRNA expression of* Ido1*,* Kmo*, and* Kynu* but not of* Tdo*,* Kat1*, or* Haao* (3-HOA) (Figure 2). There was a significant effect of stress on amygdalar* Ido1* mRNA expression (*F*(3,17) = 5.71, *p* = 0.007), *R* ^2^ = 0.50, and subsequent* post hoc* analysis demonstrated that a significant difference between the B group and the 1 h group existed (*p* < 0.05) but not between the B and the 2 h or 24 h groups (Figure 2). There were no significant increases or decreases at the 4 h (*t*(10) = 0.19, *p* = 0.86, and *R* ^2^ = 0.003) or 6 h (*t*(10) = 0.61, *p* = 0.56, and *R* ^2^ = 0.04) time point with regard to* Ido1* mRNA expression (Figure 2). Interestingly, acute restraint stress also produced a trending increase in* Kmo *(*t*(7) = 2.01, *p* = 0.09, and *R* ^2^ = 0.36) and a significant increase in* Kynu *(*t*(7) = 3.03, *p* = 0.02, and *R* ^2^ = 0.57) mRNA expression at the 4 h time point (Figure 2). There were no differences in* Kmo* mRNA expression at the 1 h, 2 h, 24 h (*F*(3,17) = 1.80, *p* = 0.19, and *R* ^2^ = 0.24), or 6 h time point (*t*(10) = 0.01, *p* = 0.99, and *R* ^2^ = 0.000009), compared to their time-matched basal controls. Likewise,* Kynu* mRNA expression at the 1 h, 2 h, 24 h (*F*(3,16) = 2.63, *p* = 0.09, and *R* ^2^ = 0.33), and 6 h time points (*t*(7) = 0.17, *p* = 0.87, and *R* ^2^ = 0.004) remained the same as time-matched controls (Figure 2).

At all time points examined (1 h, 2 h, and 24 h; 4 h; 6 h), there were no significant changes compared to time-matched basal controls (B, 4B, and 6B, resp.) with* Tdo *(*F*(3,19) = 0.94, *p* = 0.44, and *R* ^2^ = 0.13; *t*(10) = 0.41, *p* = 0.69, and *R* ^2^ = 0.02; *t*(10) = 0.78, *p* = 0.45, and *R* ^2^ = 0.06),* Kat1 *(*F*(3,18) = 0.43, *p* = 0.74, and *R* ^2^ = 0.07; *t*(10) = 0.44, *p* = 0.67, and *R* ^2^ = 0.02; *t*(10) = 0.87, *p* = 0.41, and *R* ^2^ = 0.07), and* Haao *(*F*(3,17) = 0.89, *p* = 0.47, and *R* ^2^ = 0.14; *t*(10) = 0.85, *p* = 0.41, and *R* ^2^ = 0.07; *t*(10) = 1.03, *p* = 0.33, and *R* ^2^ = 0.10) gene expression (Figure 2).

### 3.3. Acute Psychological Stress Does Not Alter Expression of Kynurenine Pathway Enzymes in the Hippocampus

**Figure 3:**
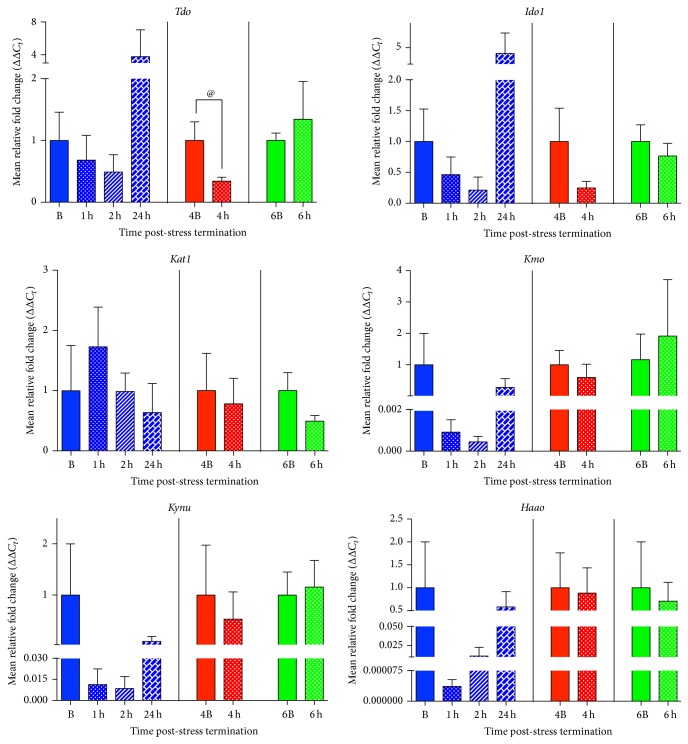
Effects of acute restraint stress on enzymes regulating the kynurenine pathway in the hippocampus. mRNA expression levels of tryptophan 2,3-dioxygenase (*Tdo*), indoleamine 2,3-dioxygenase 1 (*Ido1*), kynurenine aminotransferase (*Kat1*), kynurenine 3-monooxygenase (*Kmo*), kynureninase (*Kynu*), and 3-hydroxyanthranilate 3,4-dioxygenase (*Haao*) were examined 1–24 h post-stress termination. All data are represented as means ± SEM (*n* = 3–6/group). ^@^ *p* < 0.10 (in comparison to the 4B group).

There were no significant alterations of mRNA expression in any of the kynurenine pathway enzymes; however, hippocampal* Tdo* expression was trending towards a decrease at the 4 h time point (*t*(9) = 1.95, *p* = 0.08, and *R* ^2^ = 0.30), when compared to its time-matched control (Figure 3). There were no significant differences between the 1 h, 2 h, and 24 h time points and the B group (*F*(3,12) = 0.85, *p* = 0.49, and *R* ^2^ = 0.18) or the 6 h time point and the 6B group (*t*(9) = 0.50, *p* = 0.623, and *R* ^2^ = 0.03) for* Tdo* (Figure 3).

As mentioned above, at all time points examined (1 h, 2 h, and 24 h; 4 h; 6 h), there were no significant changes compared to time-matched basal controls (B, 4B, and 6B, resp.) in gene expression of* Ido1 *(*F*(3,14) = 1.40, *p* = 0.29, and *R* ^2^ = 0.23; *t*(9) = 1.24, *p* = 0.25; *t*(9) = 0.70, *p* = 0.50, and *R* ^2^ = 0.05),* Kat1 *(*F*(3,11) = 0.72, *p* = 0.56, and *R* ^2^ = 0.16; *t*(9) = 0.30, *p* = 0.77, and *R* ^2^ = 0.01; *t*(10) = 1.61, *p* = 0.14, and *R* ^2^ = 0.21),* Kmo *(*F*(3,15) = 1.13, *p* = 0.37, and *R* ^2^ = 0.18; *t*(7) = 0.65, *p* = 0.54, and *R* ^2^ = 0.06; *t*(8) = 0.43, *p* = 0.68, and *R* ^2^ = 0.02),* Kynu *(*F*(3,9) = 1.07, *p* = 0.41, and *R* ^2^ = 0.26; *t*(7) = 0.45, *p* = 0.67, and *R* ^2^ = 0.03; *t*(8) = 0.23, *p* = 0.83, and *R* ^2^ = 0.006), and* Haao *(*F*(3,11) = 1.20, *p* = 0.35, and *R* ^2^ = 0.25; *t*(9) = 0.13, *p* = 0.90, and *R* ^2^ = 0.002; *t*(8) = 0.27, *p* = 0.79, and *R* ^2^ = 0.009) (Figure 3).

### 3.4. Acute Psychological Stress Upregulates* Tdo* and Downregulates* Haao* Expression in the Hypothalamus

**Figure 4:**
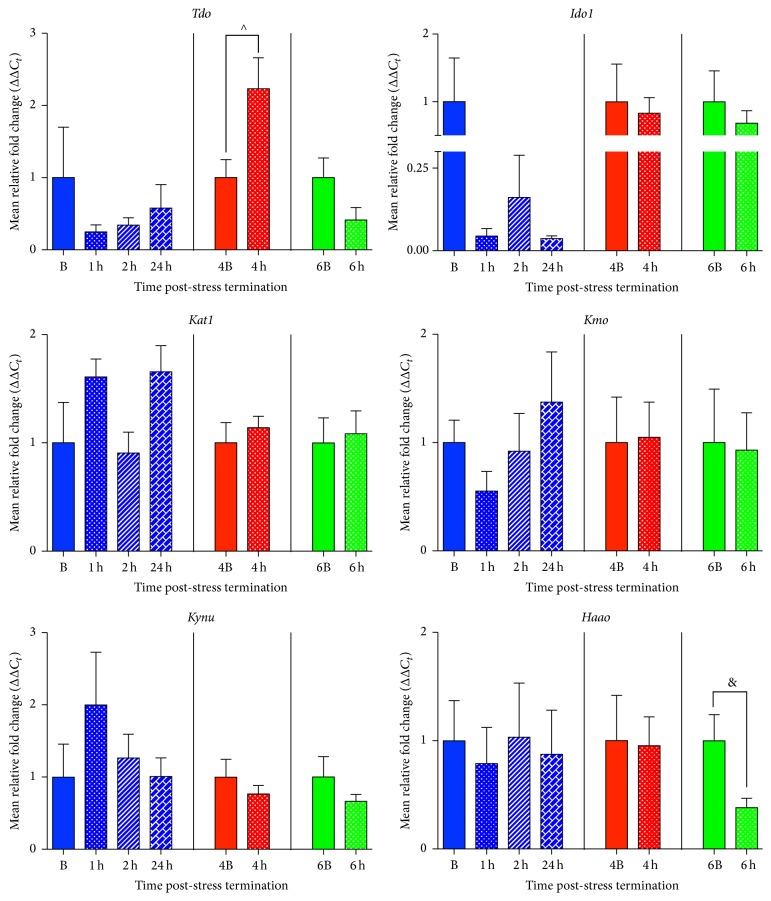
Effects of acute restraint stress on enzymes regulating the kynurenine pathway in the hypothalamus. mRNA expression levels of tryptophan 2,3-dioxygenase (*Tdo*), indoleamine 2,3-dioxygenase 1 (*Ido1*), kynurenine aminotransferase (*Kat1*), kynurenine 3-monooxygenase (*Kmo*), kynureninase (*Kynu*), and 3-hydroxyanthranilate 3,4-dioxygenase (*Haao*) were examined 1–24 h post-stress termination. All data are represented as means ± SEM (*n* = 3–6/group). ^∧^ *p* < 0.05 (in comparison to the 4B group); ^&^ *p* < 0.05 (compared to 6B group).

120 min of restraint stress significantly altered hypothalamic mRNA expression of* Tdo* and* Haao* but not of* Ido1*,* Kat1*,* Kynm*, or* Kynu* (Figure 4).* Tdo* mRNA levels were significantly elevated in the hypothalamus at the 4 h time point (*t*(6) = 2.48, *p* = 0.05, and *R* ^2^ = 0.51) but not at the 1 h, 2 h, and 24 h (*F*(3,17) = 0.79, *p* = 0.52, and *R* ^2^ = 0.12) or 6 h time point (*t*(8) = 1.81, *p* = 0.11, and *R* ^2^ = 0.29) (Figure 4). Furthermore, even though* Haao* mRNA expression was not significantly altered at the 1 h, 2 h, or 24 h time point (*F*(3,17) = 0.07, *p* = 0.97, and *R* ^2^ = 0.01) or the 4 h time point (*t*(9) = 0.10, *p* = 0.93, and *R* ^2^ = 0.001), it was significantly downregulated 6 h post-stress termination (*t*(8) = 2.41, *p* = 0.04, and *R* ^2^ = 0.42) (Figure 4).

Alterations in* Kat1* mRNA expression trended toward significance between the 1 h, 2 h, and 24 h and its time-matched basal control (*F*(3,17) = 2.51, *p* = 0.09, and *R* ^2^ = 0.31), but* post hoc* analysis showed that this was not between the basal groups and any of the stress groups but between the stress groups; furthermore, there were no changes at the 4 h or 6 h post-stress termination time point with* Kat1* mRNA expression compared to their time-matched basal controls (*t*(8) = 0.66, *p* = 0.53, and *R* ^2^ = 0.05; *t*(8) = 0.25, *p* = 0.81, and *R* ^2^ = 0.008). There were also no significant differences with regard to* Ido1 *(*F*(3,17) = 1.61, *p* = 0.22, and *R* ^2^ = 0.22; *t*(7) = 0.31, *p* = 0.77, and *R* ^2^ = 0.01;*t*(8) = 0.65, *p* = 0.54, and *R* ^2^ = 0.05),* Kmo *(*F*(3,20) = 1.10, *p* = 0.37, and *R* ^2^ = 0.14; *t*(10) = 0.09, *p* = 0.93, and *R* ^2^ = 0.0009; *t*(10) = 0.11, *p* = 0.91, and *R* ^2^ = 0.001), or* Kynu *(*F*(3,19) = 0.92, *p* = 0.45, and *R* ^2^ = 0.13); 4 h (*t*(10) = 0.86, *p* = 0.41, and *R* ^2^ = 0.07); 6 h (*t*(9) = 1.03, *p* = 0.33, and *R* ^2^ = 0.10) hypothalamic mRNA expression at any time point examined compared to time-matched basal controls (Figure 4).

### 3.5. Acute Psychological Stress Downregulates* Kynu* Expression in the Medial Prefrontal Cortex

**Figure 5:**
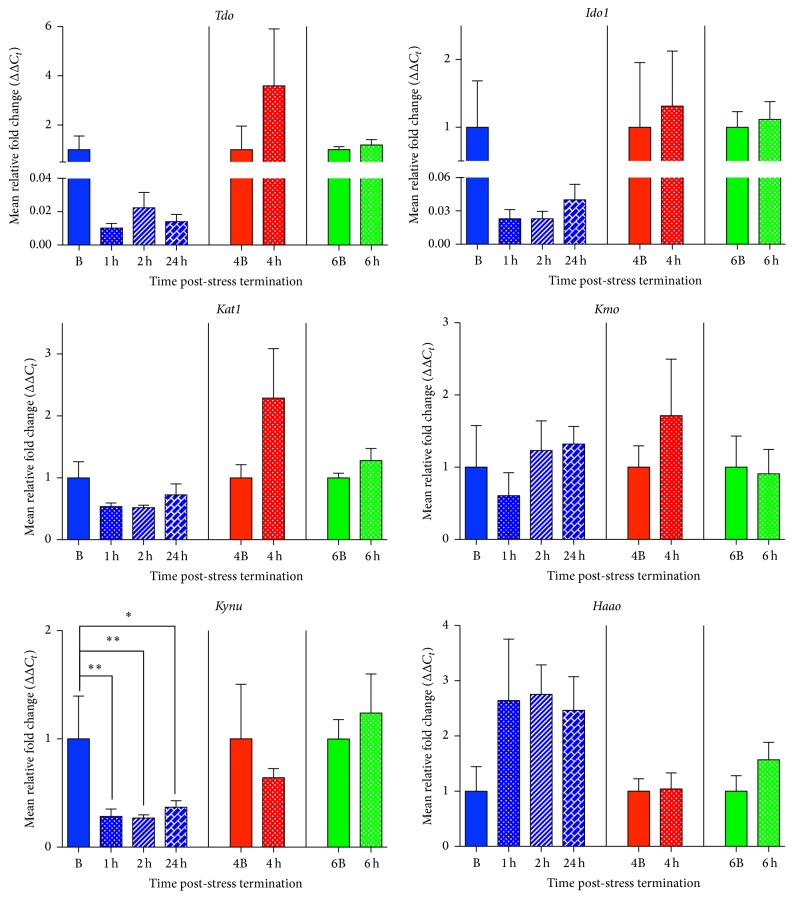
Effects of acute restraint stress on enzymes regulating the kynurenine pathway in the medial prefrontal cortex. mRNA expression levels of tryptophan 2,3-dioxygenase (*Tdo*), indoleamine 2,3-dioxygenase 1 (*Ido1*), kynurenine aminotransferase (*Kat1*), kynurenine 3-monooxygenase (*Kmo*), kynureninase (*Kynu*), and 3-hydroxyanthranilate 3,4-dioxygenase (*Haao*) were examined 1–24 h post-stress termination. All data are represented as means ± SEM (*n* = 3–6/group). ^*∗*^ *p* < 0.05 (in comparison to the B group); ^*∗∗*^ *p* < 0.01 (compared to 3B group).

120 min of acute restraint stress significantly altered mRNA expression of* Kynu* but not of* Tdo*,* Ido1*,* Kat1*,* Kmo*, or* Haao* in the medial prefrontal cortex (Figure 5). Restraint stress altered* Kynu* mRNA expression between the 1 h, 2 h, and 24 h groups and their time-matched control B (*F*(3,16) = 5.70, *p* = 0.008, and *R* ^2^ = 0.52), and* post hoc* analysis revealed that* Kynu* mRNA was downregulated at 1 h (*p* < 0.01), 2 h (*p* < 0.01), and 24 h (*p* < 0.05), in comparison to the B group. This decrease was not present at the 4 h (*t*(7) = 0.62, *p* = 0.55, and *R* ^2^ = 0.05) or 6 h post-stress (*t*(10) = 0.60, *p* = 0.56, and *R* ^2^ = 0.03) time point, when compared to their basal controls (Figure 5).

Despite trending differences in* Tdo *(*F*(3,19) = 2.88, *p* = 0.06, and *R* ^2^ = 0.31) expression between the B, 1 h, 2 h, and 24 h groups,* post hoc* analysis revealed that* Tdo* expression was not significantly different between the 1 h, 2 h, and 24 h post-stress termination groups when compared to their time-matched basal control. Furthermore, there were no differences between the 4 h (*t*(10) = 1.03, *p* = 0.33, and *R* ^2^ = 0.10) and 6 h (*t*(10) = 0.74, *p* = 0.48, and *R* ^2^ = 0.05) termination groups when compared to their time-matched basal controls. For the other enzymes in the kynurenine pathway, at all time points examined (1 h, 2 h, and 24 h; 4 h; 6 h), there were no significant changes compared to time-matched basal controls (B, 4B, and 6B, resp.) in gene expression of* Ido1 *(*F*(3,18) = 2.33, *p* = 0.11, and *R* ^2^ = 0.28; *t*(10) = 0.25, *p* = 0.81, and *R* ^2^ = 0.006; *t*(10) = 0.33, *p* = 0.75, and *R* ^2^ = 0.01),* Kat1 *(*F*(3,19) = 1.79, *p* = 0.18, and *R* ^2^ = 0.22; *t*(9) = 1.42, *p* = 0.19, and *R* ^2^ = 0.18; *t*(10) = 1.32, *p* = 0.22, and *R* ^2^ = 0.15),* Kmo *(*F*(3,15) = 0.88, *p* = 0.48, and *R* ^2^ = 0.15; *t*(7) = 1.06, *p* = 0.33, and *R* ^2^ = 0.14; *t*(10) = 0.17, *p* = 0.87, and *R* ^2^ = 0.003), and* Haao *(*F*(3,16) = 0.81, *p* = 0.51, and *R* ^2^ = 0.13; *t*(7) = 0.12, *p* = 0.91, and *R* ^2^ = 0.002; *t*(8) = 1.34, *p* = 0.22, and *R* ^2^ = 0.18) (Figure 5).

## 4. Discussion

Our results are the first to show that acute stress alters gene expression of enzymes that regulate the kynurenine pathway in a regionally dependent manner (Figure 1). Specifically, we showed that in the amygdala there is an increase in* Ido1*,* Kmo*, and* Kynu* mRNA expression following acute stress exposure (Figure 2). Interestingly, these increases occur along the pathway in a temporal manner, with* Ido1* mRNA expression coming up 1 h post-stress termination followed by* Kmo* and* Kynu* mRNA expression increases at 4 h post-stress termination (Figure 1). The only other enzyme that was increased by acute stress exposure was* Tdo* mRNA expression in the hypothalamus (Figure 4). In addition to these increases, it was also found that within the hypothalamus there was a delayed decrease in* Haao* mRNA expression (Figure 4). Decreases in the gene expression of enzymes in the kynurenine pathway were also found in the medial prefrontal cortex, specifically in* Kynu* mRNA expression, which was found to decrease at 1 h, 2 h, and 24 h post-stress termination time points (Figure 5). Finally, there were essentially no alterations from stress exposure on kynurenine pathway enzymes in the hippocampus, aside from a trending decrease in* Tdo* mRNA expression (Figure 3). Taken altogether this work showed that acute stress induces temporal and regional alterations in mRNA levels of enzymes along the kynurenine pathway; specifically, enzymes that promote the excitotoxic NMDAR agonist quinolinic acid are upregulated in the amygdala following acute stress exposure, and quinolinic pathway enzymes are decreased in the medial prefrontal cortex (Figure 1). These regional differences have been highlighted before, both in terms of acute stress-induced inflammation [16] and also in kynurenine pathway metabolites in response to chronic stress [26, 27]. Furthermore, given the ability of quinolinic acid to act as a NMDAR agonist and thus potentially increase excitability, these regional differences in the kynurenine pathway may somewhat underlie the regional changes in neuronal activity observed as a result of chronic stress or psychiatric illness, such as depression, specifically, hyperexcitable amygdala, and hypoexcitable cortex [1, 4–10].

It should be noted that there is some variability in the mRNA expression data. In spite of this, there were significant changes in enzymes, such that along the QUIN neurotoxic arm of the kynurenine pathway several enzymes are increased within the amygdala, while a downregulation of mRNA expression of these enzymes was seen in the medial prefrontal cortex, following exposure to acute stress (Figure 1). It is unclear what could be cause of this variability; however, it has been previously reported that genes that are regulated by stress or inflammation exhibit a high degree of expression variability [26, 28]. It is possible that this variability of gene expression is caused by differences in gene expression regulators (e.g., transcription factors, upstream activators) across individuals [27, 28].

As inflammation is known to induce enzymes along the kynurenine pathway, specifically, upregulation of IDO1 and enzymes that increase QUIN, a neurotoxic kynurenine metabolite [1, 13, 29], this regional specificity in response to acute restraint stress is aligned with recent work from our group demonstrating that acute stress produces regional effects on inflammatory mediators in the brain. Specifically, we found that acute restraint stress leads to an increase of inflammatory mediators in circulation and the amygdala but a decrease in the medial prefrontal cortex [16]. These regionally specific changes in inflammatory cytokine levels following acute stress parallel, to some degree, the region-specific changes we found herein. Given the ability of inflammatory cytokines to modulate tryptophan metabolism along the kynurenine pathway, it seems quite plausible that stress modulates the expression of these enzymes in a regional manner through the regulation of inflammatory mediators. Consistent with this, previous reports have demonstrated that glucocorticoids and inflammation can modulate the kynurenine pathway [12, 29, 30] and these data suggest that this may occur, in part, through alterations in the expression of the enzymes involved in the kynurenine metabolism. Whole brain alterations of kynurenine metabolites and enzymes following stress exposure require TNF-*α* and IFN*γ* [17]. As we have recently shown that acute psychological stress reliably increases TNF-*α* levels in the amygdala [16], it is quite likely that stress-induced TNF-*α* signalling mediates the documented changes here in enzymes involved in kynurenine metabolism. Having established this pattern, future work will seek to determine the discrete role of both TNF-*α* and glucocorticoids in this process.

Given that the hippocampus has traditionally been studied as a region highly sensitive to the effects of stress, it is interesting to note that this was the only brain region examined in which we did not find any significant changes in the expression of enzymes involved in kynurenine metabolism. This is consistent with the apparent lack of effect we previously reported of acute stress on inflammatory mediators in the hippocampus [16] and parallels studies demonstrating a lack of effect of chronic stress on downstream by-products of kynurenine metabolism within the hippocampus [31, 32]. As our analysis did not subdivide the hippocampus into distinct regions (e.g., CA1, CA3, and dentate gyrus), these data do not preclude the possibility of discrete subregion changes in these enzymes within the hippocampus, a hypothesis which is consistent with other data indicating that there are differences in cytokine responses within hippocampal subregions [33].

With regard to cellular sources of kynurenine metabolites, there are numerous reports that have shown that KYNA producing enzymes are primarily localized to astrocytes in the brain, while QUIN producing enzymes are primarily localized to microglia [1, 24]. As it has been reported that stress can increase microglial activation [34, 35], it is possible that these observed effects of stress on QUIN producing enzymes are due to increased microglial activation and the subsequent upregulation of gene transcription within these cells. Future work should investigate the cell-type specific origins of kynurenine pathway metabolites and enzymes in response to stress.

These regional differences in enzymes regulating the kynurenine pathway, in response to acute stress, could underlie changes that others have observed with chronic stress paradigms. Specifically, previous reports have documented an upregulation of QUIN pathway metabolites in subcortical regions (i.e., the amygdala) and downregulation of these metabolites in the cortex (cingulate cortex) [31, 32], which parallels the increase and decrease we documented of enzymes in these cascades, respectively. The functional relevance of these changes has yet to be established, but given that QUIN can activate NMDA channels and promote excitotoxicity, it is interesting to note that biochemical indications of hyperexcitability and structural damage have been found within the amygdala following exposure to chronic stress and in depression [36].

To summarize, these data present the first investigation of the effects of acute stress on regional changes in mRNA expression of enzymes regulating the kynurenine pathway. Specifically, we found changes in enzymes along the QUIN neurotoxic arm of the kynurenine pathway are increased within the amygdala, while a downregulation of mRNA expression of these enzymes was seen in the medial prefrontal cortex, following exposure to acute stress (Figure 1). More so, it seems quite plausible that these changes are a consequence of increased inflammatory mediators in discrete brain regions and could provide a mechanistic pathway linking stress-induced inflammation to alterations in cellular integrity within the brain. The data herein, demonstrating acute stress-induced changes in the gene expression of enzymes leading to kynurenine metabolites that alter excitatory signalling, may may provide insight into processes that can become dysregulated during chronic stress conditions, which could, in turn, contribute to some of the neuropathological effects documented following stress or inflammatory-related illnesses, particularly major depression.

## Figures and Tables

**Table 1:** Primer sequences used for control and kynurenine pathway enzymes.

Name	Forward 5′-3′	Reverse 5′-3′
Control primers		
*Rplp2* [16]	CGC TAC GTT GCC TCT TAT CT	GCC CAC GCT GTC TAG TAT TT
*B2m* [16]	CAG TTC CAC CCA CCT CAA ATA G	GTG TGA GCC AGG ATG TAG AAA G

Kynurenine pathway enzyme primers		
*Tdo*	GAA CTT CCT CTC CTC CTT AGA	GGC CAT TCA CAC ACT CAT TA
*Ido1*	AGG AAC AGA TGG CAG AGT	CAG TCG TCG TTC ACC TTT AC
*Kat1*	CAG ACA TCT CAG ACT TCA AGA G	CCA CCA AGC CCA TGT TT
*Kmo*	TCC ACT TTC ATC CCT CTC TAT	GAG TCC TCT GTT TAT CAC CTT T
*Kynu*	CAG ACT GCT TAC TGC CAT AC	CCC AGT GTG TGA GAT TTA CTT
*Haao*	TGA TTG AGA GAA GGC GAA TG	CCT TAC AGT GGA ACC ATT TCT

**Table 2:** Circulating corticosterone.

Time post-stress termination	Plasma corticosterone (ng/mL)
B	79.53 ± 21.29
1 h	80.94 ± 21.09
2 h	168.40 ± 74.35

4B	63.97 ± 4.27
4 h	112.00 ± 24.25

**6B**	248.90 ± 61.55^**∗**^
6 h	267.90 ± 99.53

24	33.74 ± 7.59

Effects of acute psychological restraint stress on plasma corticosterone (ng/mL) concentrations at varying times post-stress termination. All data are represented as means ± SEM (*n* = 5-6/group). ^*∗*^ *p* < 0.05 compared to B group.
